# Strip width ratio expansion with lowered N fertilizer rate enhances N complementary use between intercropped pea and maize

**DOI:** 10.1038/s41598-020-76815-7

**Published:** 2020-11-17

**Authors:** Falong Hu, Yan Tan, Aizhong Yu, Cai Zhao, Zhilong Fan, Wen Yin, Qiang Chai, Weidong Cao

**Affiliations:** 1 Gansu Provincial Key Laboratory of Arid Land Crop Science, Lanzhou, 730070 China; 2 College of Agronomy, Gansu Agricultural University, Lanzhou, 730070 China; 3 College of Forestry, Gansu Agricultural University, Lanzhou, 730070 China; 4 Institute of Agricultural Resources and Regional Planning, Chinese Academy of Agricultural Sciences, Beijing, 100081 China

**Keywords:** Plant sciences, Agroecology

## Abstract

Maize (*Zea may*s L.)/pea (*Pisum sativum* L.) strip intercropping is considered a promising cropping system to boost crop productivity. The 3-year (2009–2011) field experiment was conducted at Wuwei, northwest China, with two maize to pea strip width ratios (80:80 cm and 120:80 cm), each under three N fertilizer rates (0, 90 and 135 kg N ha^−1^ for pea, and 0, 300, and 450 kg N ha^−1^ for maize). The results showed that expanding maize to pea strip width ratio from 80:80 cm to 120:80 cm coupled with a reduction of N fertilizer rate intensified N competition and improved N compensation. The apparent N recovery and N utilization efficiency of intercropped pea with strip width ratio of 120:80 cm were increased by 8.0% and 8.9% compared to strip width ratio of 80:80 cm. Compared to high N rate, the two indicators of intercropped pea with lowered N rate were increased by 10.0% and 6.0%. For intercropped maize, the two indicators were increased by 6.8% and 5.1%, with strip width ratio of 120:80 cm compared to 80:80 cm. Also, they were improved by 9.7% and 11.5%, with lowered N rate compared to high N rate. Consequently, the grain yield of pea and maize in the 120:80 cm pattern was improved by 11.9% and 7.7% compared to 80:80 cm. We concluded that expanding maize to pea strip ratio coupled with N fertilizer reduction can optimize N complementary use.

## Introduction

One of the greatest challenge for global agriculture is to produce sufficient quantity of food, fiber and biofuels to meet the need of the fast growing human population on the planet^1^. There exists a large ‘yield gap’ in many field crops. To fulfill the gap, farmers typically apply great amounts of N fertilizer^2^. This has doubled crop yields in many areas from the middle twentieth to the early twenty first century^3^. However, an estimate that about 17–41% of the applied N fertilizer was overused^4^. The excessive use of N fertilizer rises N_2_O emission through denitrification and increases NO_3_^−^ loss though leaching, causing higher carbon footprint of agricultural products^5^. Also, it result in a series of immeasurable damages, like biodiversity loss, soil degradation, ground water pollution, and global climate change^6^. Hence, closing yield gaps must consider well of N efficient use^7^.

Multiple cropping systems are considered an efficient strategy to improve crop productivity while with lower N application^7^. Specially for intercropping, where two or more crops growing simultaneously on a same field, is remarkable at resource utilization^8^. In this cropping system, different species behave distinct biological properties. The combination of such diversified crops result in optimal canopy structure, leading to promotion of radiation interception^9^. Furthermore, the combination generates root distribution differentiation, allowing special and temporal use of soil nutrients and water^10,11^. However, grown two or more crops in the same field at a same time period may also aggravate competition of limited resources^12^. This commonly result in yield reduction of subdominant crops^13,14^. Therefore, how to optimize interspecific interactions to maximize N utilization is vital for intercropped species.

In intercropping, an earlier-sowing crop is usually combined with a later-sowing crop. Generally, a rapid initial growth may allow the earlier-sown species to retrieve nutrients faster than the later-sown one, which prevents its predominance^15^. In maize/faba bean (*Vicia faba* L.) intercropping, an earlier-sowing of faba bean leading to a relative higher competition^16^. In durum wheat (*Triticum turgidum* L.)/winter pea (*Pisum sativum* L.) intercropping, winter pea growing faster and competed greatly than durum wheat^17^. In maize/pea intercropping, pea sown earlier and had higher competition than component maize^18^. Hence, intensify competition may increase N use efficiency of earlier-sown species. Whereas, such a competition often result in restricted growth of component crop^14^. Howbeit, competition is not persistent and will vanish immediately after harvest of earlier-sown species^13,19^. Since after, a recovery growth for subdominant crop due to aboveground canopy stretch and belowground root expansion will occur^20^. This helps the later-sown crop to reach a fully recovery from the restricted growth^18^.

Maize and pea are most abundantly-grown field crops in northwest China, where the two crops are customarily intercropped together^21^. It has been considered as a promising model for sustain food production in a sustainable manner^22^. However, how the N is used between intercropped pea and maize remain unclear. There is a need to determine the effect of interspecific interactions on N use in maize/pea intercropping. Here, the maize to pea strip width ratio and N fertilizer rate was managed to provide various effect of interspecific interactions on N use. Therefore, the primary objective of this study was to evaluate how strip width ratio and N fertilizer rate would affect N use of intercropped pea and maize. We tested the hypotheses that (1) expanding maize to pea strip width ratio with reduced N fertilizer rate could intensify the N competition and improve N compensation, and (2) intensified N competition and improved N compensation would increase N use efficiency and productivity of pea and maize. In testing the hypothesis, we determined (1) grain yield and N accumulation of sole and intercropped pea and maize, (2) N competition of pea relative to maize and N compensation of maize, and (3) apparent N recovery and N utilization efficiency of pea and maize.

## Materials and methods

### Experimental site

**Figure 1:**
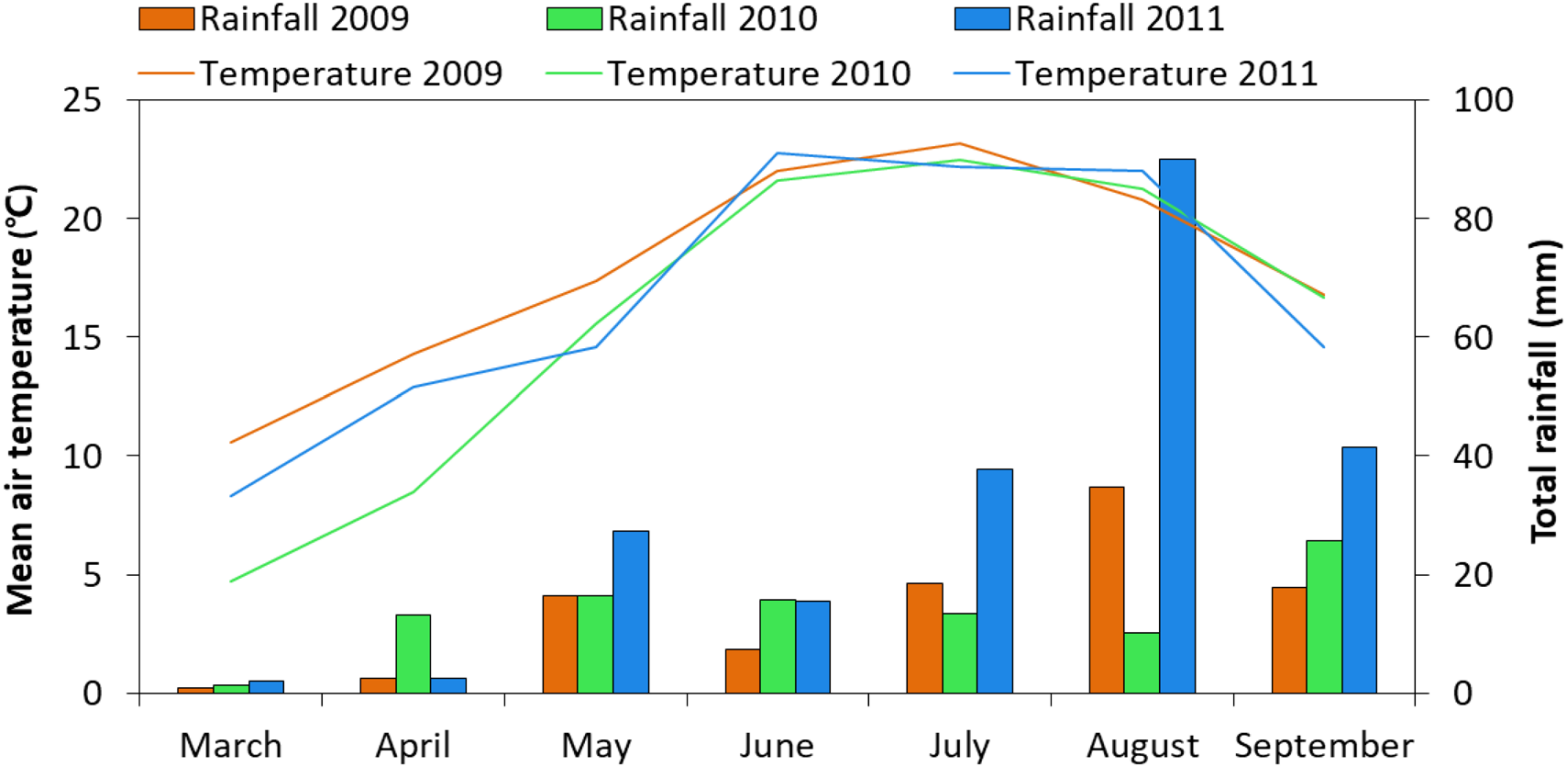
Monthly mean air temperature and total rainfall during the growing season of maize/pea intercropping in 2009, 2010, and 2011 at Wuwei experimental station, northwestern China.

Field experiments were conducted in 2009, 2010, and 2011 at the Oasis Agricultural Research Station (37° 30′ N, 103° 5′ E; 1776 m a.s.l.) located in the Hexi Corridor of northwestern China. The station is at the temperate arid zone in the hinterland of the Eurasia Continent. Soil was an Aridisol^23^. Long-term (1960–2009) annual mean temperature is 7.2 °C with accumulated temperature above 0 °C > 3513 °C and above 10 °C > 2985 °C; annual rainfall is 156 mm mostly occurring June through September (Fig. 1), while annual evaporation often exceeds 2400 mm^21^. Solar radiation is ~ 6000 MJ m^−2^, with sunshine duration of 2945 h and frost-free period of 156 days. Total nitrogen (N) was 0.78 g kg^−1^ and available N, P, and K were 25.6, 33.8, and 134.2 mg kg^−1^, respectively. The natural resources (heat, radiation, the length of the growing season) are typically suitable for intercropping.

### Experimental design

**Figure 2:**
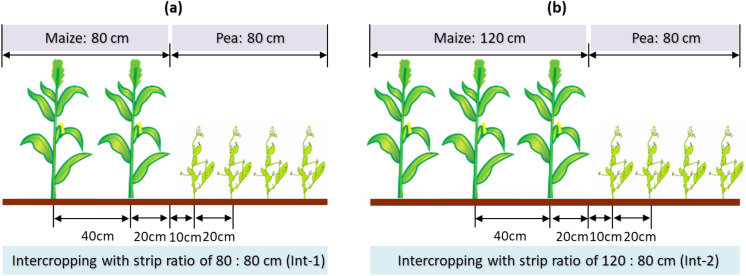
Strip structure of maize/pea intercropping with the strip ratio of (**a**) 80:80 cm, having two rows of maize and four rows of pea (Int-1), and (**b**) 120:80 cm, having three rows of maize and four rows of pea (Int-2).

The experiment was a split-plot design with three replicates. Main plot treatments included four cropping systems: sole maize, sole pea, and maize/pea intercropping with strip-width-ratio of 80:80 cm (Int-1), and of 120:80 cm for maize: pea (Int-2) (Fig. 2; Table 1). In the Int-1 relay system, pea and maize were alternated in a 160-cm wide strip, each occupying ½ of the land space, whereas in the Int-2 system, the 200-cm wide strip was shared at the ratio of 3/5 of the land area by maize and the remaining 2/5 of the land area by pea. Subplot treatments included three N fertilizer rates, i.e. N0 (the control), N1 (300 kg N ha^−1^ for maize, 90 kg N ha^−1^ for pea), and N2 (450 kg N ha^−1^ for maize, 135 kg N ha^−1^ for pea). For maize in the N1 and N2 treatments, 30% of total N was broadcasted on the soil surface and incorporated into the soil using a disk harrower prior to sowing, 60% of the total N was top-dressed at pre-tasseling stage of maize, and the remaining 10% was top-dressed at grain filling stage. Top-dressing was accomplished using a fertilizer placement drill that made a furrow 5–6 cm deep, 4–5 cm away from the maize plant and fertilizer was applied to the furrow, prior to irrigation. For pea, all the N fertilizer was applied as the base N prior to sowing. All plots received P fertilizer (in form of P_2_O_5_) at 150 kg P ha^−1^as a base fertilizer; then, for the plots without zero N fertilizer, calcium superphosphate (0–16–0 of N–P_2_O_5_–K_2_O) was applied, while for the plots with N fertilizer, diammonium phosphate (18–46–0 of N–P_2_O_5_–K_2_O) was applied. In all treatments, crop residues were removed out of the fields for animal feeding (“Supplementary information”).

**Table 1:** Information on agronomic practices of pea and maize in the sole and intercropping systems at the Wuwei Oasis Agricultural Experimental Station in northwestern China in 2009–2011.

Item	Strip width (cm)		Planting rows per strip		Planting density (plants ha^−1^)		Irrigation quota (mm)	
	Pea	Maize	Pea	Maize	Pea	Maize	Pea	Maize
Sole cropping (S)	–	–	–	–	1,800,000	90,000	165	405
Intercropping 1 (Int-1)	80	80	4	2	900,000	45,000	82.5	202.5
Intercropping 2 (Int-2)	80	120	4	3	720,000	54,000	66	243

Field pea (cv. Long-wan No. 1) was planted in early April and harvested in early July; and maize (cv. Wu-ke No. 2) was planted in late April and harvested in late September. The plot size was 38.4 m^2^ (4.8 m × 8 m) for Int-1, 45.6 m^2^ (5.7 m × 8 m) for Int-2, and 48 m^2^ (6 m × 8 m) for sole cropping. The system layout of Int-1 was 160-cm-wide strips consisting of two rows (40 cm interrow) of maize with a strip with of 80 cm, and four rows (20 cm interrow) of pea with strip width of 80 cm. While the layout of Int-2 was 200-cm-wide strips consisting of three rows (40 cm interrow) of maize with strip with of 120 cm, and four rows (20 cm interrow) of pea with strip width of 80 cm (Table 1). There were three pairs of maize-pea strips in each intercropping plot. All maize strips were mulched with plastic film to increase soil temperature and conserve soil moisture^24^. Seeding rates were based on seed germination and estimated emergence rate to target an optimal plant population of 1800,000 plants ha^−1^ for pea and 90,000 plants ha^−1^ for maize. Seeding rates for crops in Int-1 and Int-2 were calculated based on the actual land areas each crop occupied in the strips. Due to low precipitation at the testing areas (< 155 mm annually), supplemental irrigation was applied^18,25^. All plots received 120 mm of irrigation the previous fall just before soil freezing, and then various irrigation quotas during the growing season were applied to the crops by drip irrigation to satisfy the treatment requirements (Table 1). The same area based irrigation quota was implemented for both pea and maize in the sole cropping and intercropping at each irrigation event.

### Grain yield

All plots were harvested when pea and maize reached full maturity. The grains were air-dried, cleaned, and weighed for the grain yield (GY) of the sole and intercropped crops individually.

### Dry matter and N accumulation

Pea and maize plants were sampled at 15 day intervals before pea harvest and then maize plants were continuously sampled at 25 day intervals until maize harvest. The first sampling took place 15 days after maize sowing. At each sampling date, ten maize plants and four adjacent rows of pea plants (4 rows × 30-cm long) were randomly selected from each plot, cut off the whole plants on the soil surface, separate stems, leaves, and grains in the laboratory, oven-dried at 80 °C to a constant weight, and weighed for dry matter of the individual organs. Nitrogen concertation (N%) of each samples was determined using a high-performance C and N analyzer (Elementar vario MACRO cube, Germany). Nitrogen accumulation in each organ (NA, kg N ha^−1^) was calculated as: NA = *N*% × *DM*, where *N*% is the N concentration, and *DM* is plant dry matter.

### Calculation of nitrogen competition

We hypothesize that there is a certain level of competition for available soil N between intercropped maize and intercropped pea during the co-growth period. To quantify the competitiveness, we adopted the concept of between-species competitiveness^26^ and defined the term ‘competitive ratio—CR’. Thus, N competitive ratio (N-CR) of intercropped pea to maize is calculated as:

1 $$ {\text{N - CR}} = \left( {\frac{{{{NAip} \mathord{\left/ {\vphantom {{NAip} {NAsp}}} \right. \kern-\nulldelimiterspace} {NAsp}}}}{{{{NAim} \mathord{\left/ {\vphantom {{NAim} {NAsm}}} \right. \kern-\nulldelimiterspace} {NAsm}}}}} \right) \times \frac{Fm}{{Fp}} $$

where NA*ip* and *NAsp* are N accumulations of pea in intercropping and sole cropping, and *NAim* and *NAsm* are the nitrogen accumulations of maize in the two cropping system. *Fm* and *Fp* are the land proportion occupied by maize and pea in the intercropping, respectively. An N-CR value greater than 1 indicates the intercropped pea has the advantage for the available soil N; an N-CR value less than 1 means maize has the advantage; an N-CR value = 1 means intercropped pea and maize are equally competitive.

### Calculation of nitrogen compensation

When intercropped pea is harvest, intercropped maize plants could root to pea strips to take the advantage of available soil N to ‘supplement’ the growth^18^. Thus N compensation can be indicated by intercropping advantage on N accumulation of maize over the sole cropping during specific times (i.e. recovery growth stages). We adopted the term ‘compensation effect—CE’^25^ to quantify N compensation, which is calculated as:

2 $$ {\text{N - CE}} = \frac{NARim}{{NARsm}} \times \frac{1}{Fm} $$

where *NARim* and *NARsm* are N accumulation rate (NAR) of maize plants in the sole and intercropping systems. The NAR of maize is determined by the equation: NAR = (*NA*_2_ − *NA*_1_)/(*t*_2_ − *t*_1_*)*, where *NA*_*1*_ and *NA*_*2*_ are the amount of N accumulated by maize plants at the two (*t*_*1*_ and *t*_*2*_) sampling dates. A value of N-CE higher than 1.0 indicates intercropped maize has N compensation after accompanying pea harvest, while a value equal and/or lower than 1.0 indicates there is no N compensation.

### Apparent nitrogen recovery

Apparent nitrogen recovery (ANR, %), defined as per unit area N accumulation in pea and maize provided by N-fertilizer^27^, is calculated as:

3 $$ ANR = \frac{{NA_{with\;N} - NA_{without\;N} }}{{N_{rate} }} \times 100\% $$

where *NA*_*with N*_ and *NA*_*without N*_ are N accumulations in pea and maize with N application and with zero N application, and *N*_rate_ represent N-fertilizer rate.

### Nitrogen utilization efficiency

Nitrogen utilization efficiency is defined as grain yield divided by N accumulation (NutE, g g^−1^). It was calculated as Fageria and Baligar^27^:

4 $$ {\text{NutE}} = \frac{GY}{{NA}} $$

### Statistical analysis

Data were analyzed using analysis of variance with SPSS 17.0 (SPSS Institute Inc.). Replication was considered a random effect and year, cropping system, and N management system were considered fixed effects. Means were compared using Fisher’s protected least significant difference test and significance was declared at *P* ≤ 0.05, unless otherwise stated. The correlation analysis was conducted using the Pearson’s correlation of SPSS 17.0 to clarify the relations between parameters.

## Results

### Grain yield

In terms of GY of pea, there was no significant difference between N rate at 90 kg N ha^−1^ (N1) and 135 kg N ha^−1^ (N2) in both cropping sole and intercropping (Table 2). While it of intercropping with maize to pea strip width ratio of 120 cm:80 cm (Int-2) was increased by 12.7% with N0, 11.9% with N1 and 11.1% with N2, compared to intercropping with maize to pea strip width ratio of 80 cm:80 cm (Int-1). For GY of maize, no significant difference between N rate at 300 kg N ha^−1^ (N1) and 450 kg N ha^−1^ (N2) was found in both Int-1 and Int-2; but in sole cropping, it with N1 was reduced by 7.4% compared to N2 (Table 2). Besides, Int-2 had 7.7% greater GY than Int-1.

**Table 2:** Grain yield (GY) of pea and maize in the sole and intercropping under three N fertilizer rates in 2009–2011.

Cropping pattern^a^	N rate^b^	2009		2010		2011	
		Pea	Maize	Pea	Maize	Pea	Maize
		t ha^−1^					
Sole cropping	N0	2.45 g	7.76 d	2.65 f	7.70 e	2.77 e	7.01 f
	N1	3.67 de	10.88 bc	3.49 cd	11.87 c	3.55 c	11.02 d
	N2	3.56 e	12.23 b	3.70 c	13.17 b	3.55 c	11.06 d
Intercropping (Int-1)	N0	2.63 g	9.59 c	3.07 e	9.44 d	3.19 d	9.59 e
	N1	4.16 bc	14.10 a	4.24 b	14.25 ab	4.01 b	14.42 bc
	N2	4.01 cd	14.32 a	4.05 b	14.37 c	4.16 b	14.29 c
Intercropping (Int-2)	N0	3.08 f	10.76 bc	3.34 de	10.85 d	3.59 c	10.64 d
	N1	4.58 a	15.30 a	4.75 a	15.31 a	4.55 a	15.23 a
	N2	4.45 ab	15.27 a	4.66 a	14.85 a	4.48 a	15.00 ab
**Significance (P value)**							
Year (Y)		< 0.001	0.010	–	–	–	–
Cropping system (C)		< 0.001	< 0.001	< 0.001	< 0.001	< 0.001	< 0.001
N rate (N)		< 0.001	< 0.001	< 0.001	< 0.001	< 0.001	< 0.001
C × N		NS	NS	0.002	0.014	NS	NS
Y × C × N		NS	NS	–	–	–	–

Means with different letters in the same column are significantly different at *P* < 0.05 by LSD.

^a^Int-1 and Int-2 means the intercropping with strip ratio of 80:80 cm and 120:80 cm for maize: pea, respectively.

^b^N0 is the control at N = 0 kg ha^−1^. N1 and N2 for pea represents the N fertilizer rate at 90 kg N ha^−1^ and 135 kg N ha^−1^, and for maize represents N rate at 300 kg N ha^−1^ and 450 kg N ha^−1^.

### Nitrogen accumulation

**Figure 3:**
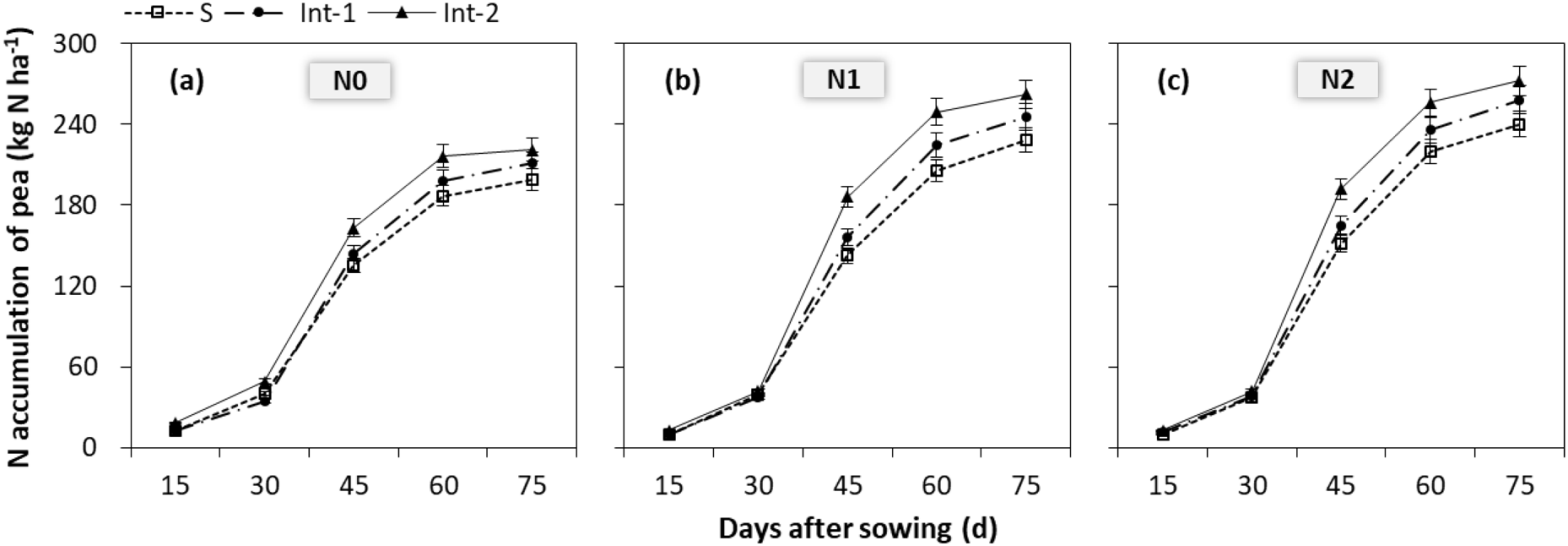
Nitrogen accumulation of pea with (**a**) N0, (**b**) N1 and (**c**) N2 in three cropping systems. S, sole cropping; Int-1, intercropping with strip width ratio of 80:80 cm for maize:pea; Int-2, intercropping with strip width ratio of 120:80 cm for maize:pea. N0, N1, and N2 represent the N fertilizer rate at 0, 90 and 135 kg N ha^−1^ for the pea crop. 15, 30, 45, 60, and 75 day after sowing are respectively at the VE, V3, V8, V12 and V16 stage of maize. The error bars are standard error of the means (*n* = 9).

The NA in pea plants increased with growth stage processing, and reached the highest value until maturity (Fig. 3). Year × cropping system × N rate interaction had no significant (*P* = 0.163) effect on total NA of pea, but cropping system (*P* < 0.001), N rate (*P* < 0.001) and cropping system × N rate interaction (*P* = 0.009) significantly affected it. In sole cropping, the NA of pea with N1 was reduced by 4.8% compared to N2. While in int-1 and int-2, there was no significant difference of NA between N1 and N2. In addition, it in Int-2 was increased by 5.7% compared to Int-1.

**Figure 4:**
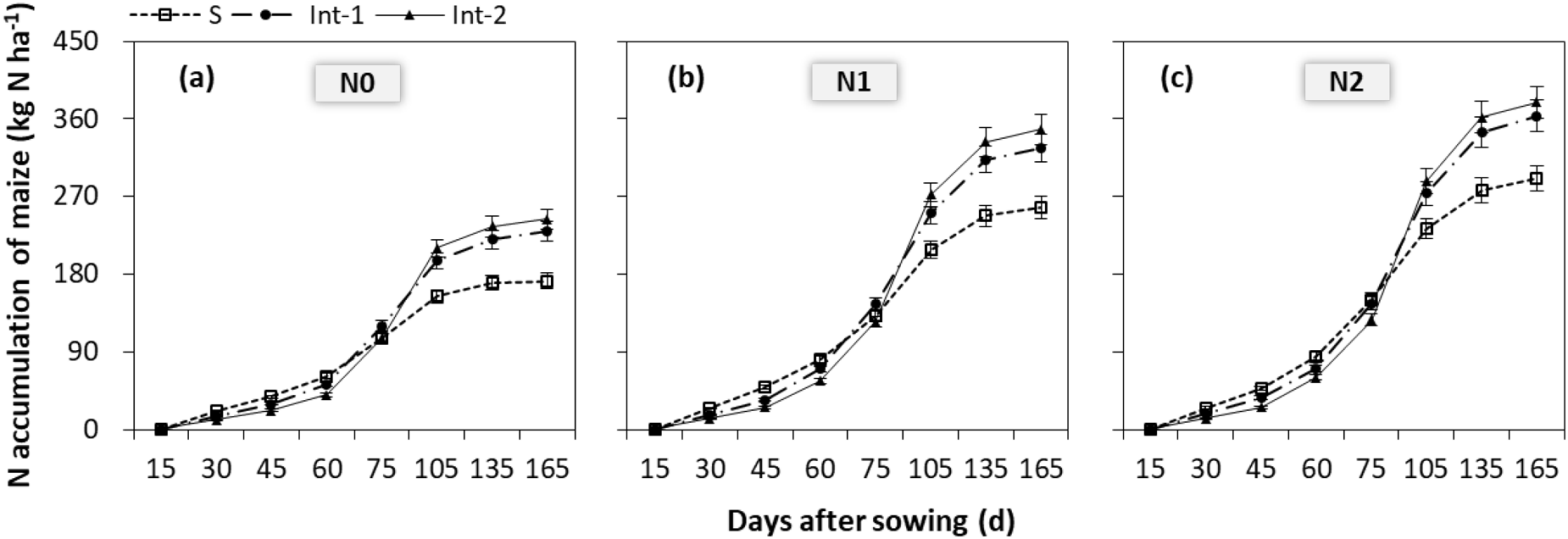
Nitrogen accumulation of maize with (**a**) N0, (**b**) N1 and (**c**) N2 in three cropping systems. S, sole cropping; Int-1, intercropping with strip width ratio of 80:80 cm for maize:pea; Int-2, intercropping with strip width ratio of 120:80 cm for maize:pea. N0, N1 and N2 represent the N fertilizer applied at 0, 300 and 450 kg N ha^−1^ for the maize. 15, 30, 45, 60, 75, 105, 135 and 165 day after sowing are respectively at the VE, V3, V8, V12, V16, R1, R4 and R6 stage of maize. The error bars are standard errors of the means (*n* = 9).

The dynamic of NA in maize plants varied greatly from before-pea-harvest (i.e. before 75 days) to after-pea-harvest (i.e. after 75 days). Generally, NA of maize in sole cropping was greater than that in intercropping before 75 days. While, after 75 days, it in sole cropping was lower than intercropping (Fig. 4). This indicated that there was a competitive disadvantage of maize plants before-pea-harvest, while a compensation effect after-pea-harvest. At maturating (i.e. 165 days), cropping system, N rate and cropping system × N rate interaction significantly (*P* < 0.001) affected NA of maize, but not did by year × cropping system × N rate interaction (*P* = 0.348). In sole cropping, the NA of maize with N1 was reduced by 12.0% compared to N2. Whereas, in int-1 and int-2, there was no significant difference of NA between N1 and N2. In addition, Int-1 and Int-2 increased the NA by 27.5 and 34.6% compared to sole maize, respectively. And Int-2 increased the NA by 5.5% compared to Int-1.

### Nitrogen competition

**Figure 5:**
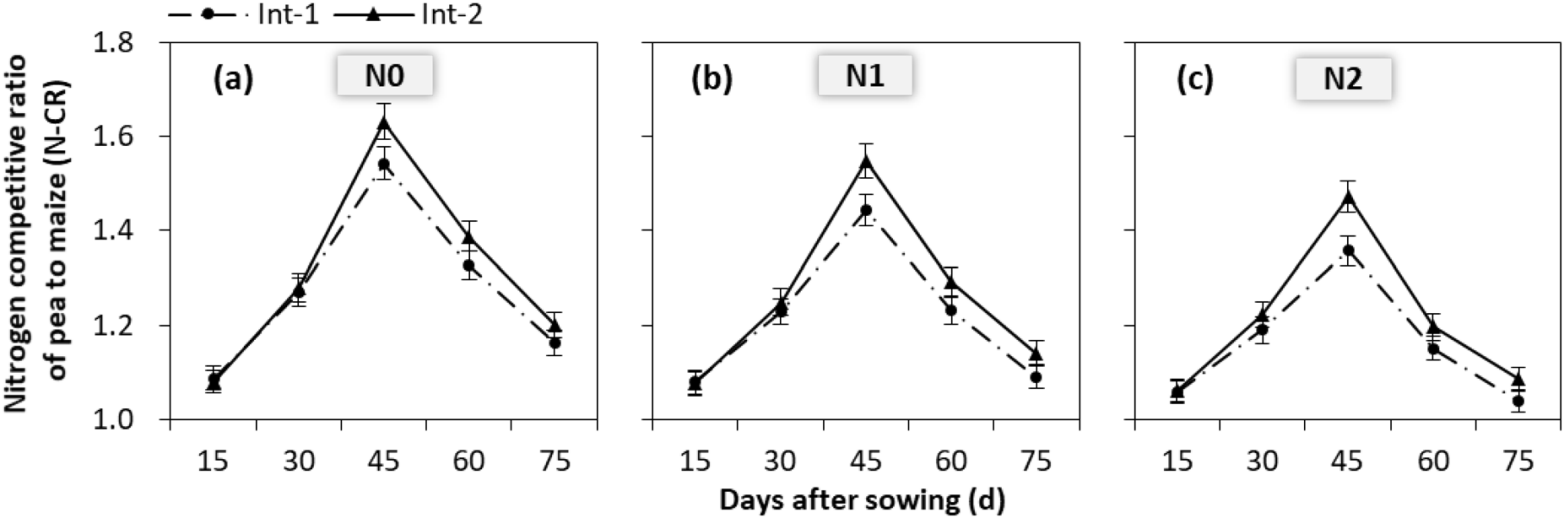
Seasonal dynamics of nitrogen competitive ratio (N-CR) of pea to maize with (**a**) N0, (**b**) N1 and (**c**) N2 in the two intercropping systems. Int-1, intercropping with strip width ratio of 80:80 cm for maize:pea; Int-2, intercropping with strip width ratio of 120:80 cm for maize:pea. N0, N1 and N2 mean the N fertilizer applied at 0, 90 and 135 kg N ha^−1^ for pea, and at 0, 300 and 450 kg N ha^−1^ for maize, respectively. 15, 30, 45, 60, and 75 day after sowing are respectively at the VE, V3, V8, V12 and V16 stage of maize. The error bars are standard errors of the means (*n* = 9).

Generally, N-CR increased from 15 days after maize sowing (VE stage), reached the peak at 45 days after maize sowing (V8 stage), and declined until pea senescence (Fig. 5, Supplementary file 1). The magnitude of N-CR varied greatly with cropping system and N rate since 45 days after maize sowing. At 45 days, the N-CR of Int-2 was increased by 7.1% compared to Int-1 (Table 2). And it with N1 was increased by 5.8% compared to N2. At 60 days after maize sowing, it of Int-2 was increased by 4.5% compared to Int-1; and it with N1 was increased by 7.5% compared to N2. Similarly, at 75 days after maize sowing, it was increased by 4.1% with Int-2 compared to Int-1, and by 5.0% with N1 compared to N2.

### Nitrogen compensation

The N-CE was assessed during three reproductive growth stages, i.e. 75–105 (V16–R1), 105–135 (R1–R4), and 135–165 days after maize sowing (R4–R6). At 75–105 days after maize sowing, the N-CE of Int-2 was improved by 8.2% compared to Int-1; and it with N1 was improved by 5.7% compared to N2 (Table 3). At 105–135 days after maize sowing, it of Int-2 was increased by 10.3% compared to Int-1. And it with N1 was increased by 5.5% compared to N2. At 135–165 days after maize sowing, in 2009, it with N1 was improved by 5.9% in Int-1, and by 10.7% in Int-2, compared to N2. Besides, Int-2 had 11.5% greater N-CE than Int-1. In 2010, it of Int-2 was increased by 9.6% compared to Int-1; and it with N1 was increased by 7.1% compared to N2. In 2011, it with N1 was improved by 11.2% in Int-1, and by 5.6% in Int-2, compared to N2. In addition, Int-2 had 9.5% greater N-CE than Int-1.

**Table 3:** Nitrogen compensation effect (N-CE) of intercropped maize over sole maize during the three recovery growth stages under three N levels in 2009–2011.

Cropping system^a^	N rate^b^	2009			2010			2011		
		75–105 ^c^	105–135	135–165	75–105	105–135	135–165	75–105	105–135	135–165
Intercropping (Int-1)	N0	1.45 b	1.14 e	1.02 e	1.40 b	1.25 e	1.04 e	1.39 b	1.20 d	1.06 e
	N1	1.22 d	1.26 cd	1.46 bc	1.30 c	1.39 c	1.51 b	1.23 d	1.33 b	1.48 b
	N2	1.16 e	1.22 d	1.38 c	1.22 d	1.31 d	1.39 c	1.16 e	1.24 c	1.33 c
Intercropping (Int-2)	N0	1.57 a	1.31 c	1.13 d	1.55 a	1.39 c	1.14 d	1.53 a	1.27 c	1.13 d
	N1	1.32 c	1.45 a	1.67 a	1.38 b	1.50 a	1.62 a	1.31 c	1.43 a	1.60 a
	N2	1.24 d	1.38 b	1.51 b	1.32 c	1.45 b	1.54 b	1.25 d	1.33 b	1.51 b
**Significance (P value)**										
Year (Y)		< 0.001	< 0.001	0.039	–	–	–	–	–	–
Cropping system (C)		< 0.001	< 0.001	< 0.001	< 0.001	< 0.001	< 0.001	< 0.001	< 0.001	< 0.001
N rate (N)		< 0.001	< 0.001	< 0.001	< 0.001	< 0.001	< 0.001	< 0.001	< 0.001	< 0.001
C × N		NS	NS	0.030	NS	NS	NS	NS	NS	0.002
Y × C × N		NS	NS	0.003	–	–	–	–	–	–

Means with different letters in the same column are significantly different at *P* < 0.05 by LSD.

^a^Int-1 and Int-2 means the intercropped maize grown in the 80:80 cm and 120:80 cm strips, respectively.

^b^N0, N1 and N2 represents the N fertilizer rate at 0, 300 and 450 kg N ha^−1^, respectively.

^c^N-CE was assessed during the three reproductive growth stages (i.e., 75–105, 105–135, and 135–165 days after sowing, or V16–R1, R1–R4, and R4–R6 of maize, respectively).

### Apparent nitrogen recovery

Intercropping pea and maize achieved higher ANR than sole cropping (Table 4, Supplementary file 2). For ANR of pea, it with N1 was enhanced by 8.0% in sole cropping, 10.0% in Int-1 and 20.0% in Int-2, compared to N2. Besides, it in Int-1 and Int-2 was enhanced by 15.9 and 25.2% compared to sole cropping. Int-2 had 8.0% greater ANR than Int-1. For ANR of maize, it with N1 was enhanced by 7.0% in sole cropping, 7.9% in Int-1 and 11.4% in Int-2, compared to N2. In addition, it in Int-1 and Int-2 was enhanced by 16.2 and 24.0% compared to sole cropping. And Int-2 had 6.8% greater ANR than Int-1.

**Table 4:** Apparent N recovery (ANR) and N utilization efficiency (NutE) of pea and maize in the sole and intercropping systems under three N fertilizer rates across 2009 to 2011.

Cropping pattern^a^	N rate^b^	Apparent nitrogen recovery (%)		Nitrogen utilization efficiency (%)	
		Pea	Maize	Pea	Maize
Sole cropping	N0	–	–	13.2 e	42.6 e
	N1	33.1 cd	28.4 c	15.6 cd	43.7 d
	N2	30.6 d	26.5 d	15.0 d	41.7 f
Intercropping (Int-1)	N0	–	–	13.6 e	45.8 c
	N1	38.4 b	31.9 b	16.4 bc	46.8 b
	N2	34.9 c	29.6 c	15.4 cd	42.2 ef
Intercropping (Int-2)	N0	–	–	14.8 d	48.9 a
	N1	45.6 a	35.0 a	17.9 a	49.0 a
	N2	38.0 b	31.4 b	16.7 b	43.8 d
**Significance (P value)**					
Year (Y)		NS	0.024	< 0.001	< 0.001
Cropping system (C)		0.001	< 0.001	< 0.001	< 0.001
N rate (N)		0.047	< 0.001	< 0.001	< 0.001
C × N		< 0.001	< 0.001	NS	< 0.001
Y × C × N		NS	NS	NS	NS

Means with different letters in the same column are significantly different at *P* < 0.05 by LSD.

^a^Int-1 and Int-2 means the intercropping with strip ratio of 80:80 cm and 120:80 cm for maize: pea, respectively.

^b^N0 is the control at N = 0 kg ha^−1^. N1 and N2 for pea represents the N fertilizer rate at 90 kg N ha^−1^ and 135 kg N ha^−1^, and for maize represents N rate at 300 kg N ha^−1^ and 450 kg N ha^−1^.

### Nitrogen utilization efficiency

Overall, the NutE of pea and maize in intercropping was higher than that in sole cropping, and it of maize was greater than pea (Table 4, Supplementary file 3). For NutE of pea, Int-1 and Int-2 increased it by 3.5% and 12.7% compared to sole pea, respectively. The Int-2 had 8.9% greater NutE than Int-1. Among N rate treatments, N1 had the highest NutE, which was enhanced by 19.7 and 6.0% compared to N0 and N2, respectively. For NutE of maize, N1 treatment achieved the highest value, which was enhanced by 4.9% in sole cropping, 10.9% in Int-1, and 12.0% in Int-2, compared to N2. In addition, Int-1 and Int-2 enhanced it by 5.3 and 10.7% compared to sole cropping. The Int-2 had 5.1% greater NutE than Int-1.

### Correlation of N-CR and N-CE relative to NA, ANR and NutE

There was a significant (*P* ≤ 0.046) positive correlation of NA in pea plants with N-CR at 45 and 60 days after maize sowing, but not at 75 days (Table 5). This indicated that N competition of pea contributed greatly to total NA. And the contribution closely related to growth status of pea. A vigorous growth result in higher contribution, but senescence ceased the contribution. Furthermore, the significant (*P* ≤ 0.023) positive correlation of ANR and NutE of pea with N-CR indicating that N competition improved N uptake from applied fertilizer, and promoted the accumulated N to form more grains.

**Table 5:** Pearson’s correlation coefficients of N competitive ratio (N-CR) and N compensation effect (N-CE) relative to NA, ANR and NutE of pea and maize across 2009 to 2011.

Item	Sampling date (days)	Pea			Maize		
		NA^a^	ANR^a^	NutE^a^	NA	ANR	NutE
N-CR	45	0.560**	0.711**	0.567**	− 0.051	0.694**	0.574**
	60	0.392*	0.587**	0.444**	− 0.136	0.644**	0.561**
	75	0.328	0.620**	0.493**	− 0.334	0.631**	0.647**
N-CE^b^	75–105	–	–	–	− 0.225	0.685**	0.722**
	105–135	–	–	–	− 0.088	0.645**	0.669**
	135–165	–	–	–	− 0.206	0.752**	0.791**

^a^ NA, ANR and NutE represents N accumulation, apparent N recovery and N utilization efficiency, respectively.

^b^N-CE was applicable to maize pants only.

*Correlation coefficient was significant at *P* < 0.05.

**Correlation coefficient was significant at *P* < 0.01.

The NA in maize plants hardly (*P* ≥ 0.472) correlated with N-CE in each of the three growth periods, implying that any potential effect of N compensation on NA of maize might be masked by N fertilizer application (Table 5). Nevertheless, N-CE was significantly (*P* ≤ 0.011) and positively correlated with ANR and NutE of maize. This indicated that N compensation drived maize plants to acquire more N from fertilizer and promoted the accumulated N to form more grains. Moreover, there was a significant (*P* ≤ 0.046) positive correlation of N-CR with NutE and ANR of maize, signifying that N competition from pea plants might be the initial trigger for improved NutE and ANR of maize.

## Discussion

### Performance of N complementary use

Intercropping is reported with many advantages on productivity, pest and disease control, ecological services, and economic profitability^8,28,29^. The cereal-legume intercropping is considered the most effective model among various intercropping systems^30^. In this system, cereal plants can compete soil N with legume plants, resulting in depletion of N status, then stimulates the biological N_2_ fixation^31,32^. Also, the fixed N can be assimilated by cereal plants through N transfer^10^. However, other studies showed that legume had a relative higher competition than cereal when intercropped together^16,18^. In the present study, the N competitive ratio of pea relative to maize was consistently greater than 1, indicating pea is the dominant crop in the system. This was verified by the N accumulation of intercropped pea greater than sole pea, while that of intercropped maize lower than sole maize before pea harvest. Furthermore, expanding maize to pea strip width ratio and reducing N fertilizer rate significantly intensified the N competition from pea plants to component maize. Compared to intercropping with strip width ratio of 80 cm:80 cm (Int-1), N competitive ratio in strip width ratio expended (i.e. 120 cm:80 cm) intercropping (Int-2) was increased by an average of 5.2% since 45 days after maize sowing. Compared to higher N fertilizer rate at 135 kg N ha^−1^, the N competitive ratio with lowered N rate (i.e. 90 kg N ha^−1^) was increased by an average of 6.1% since 45 days after maize sowing.

The mechanism on intensified N competition due to strip width ratio expansion is not clear, but we assumed the following possibility: (1) expanding strip width ratio could promote intraspecific competition between maize plants in border and inner rows, (2) the increased intraspecific competition may enhance maize interspecific competition against component pea, (3) the increased interspecific competition may in turn strengthen competition of pea due to pea is the dominant crop; furthermore, it may also stimulate the biological N_2_ fixation of pea^18^, and (4) the increase of N uptake from maize strip and N_2_ fixation contributed to higher N accumulation in pea plants and result in higher N competition. However, the present study lack of direct verification of N trace and N transfer, make the actual outcome unclear. Therefore, further studies that focusing on N fate in maize/pea intercropping are needed.

After early-sown crop harvest, the late-sown crop can recover the impaired growth through compensation^18,25^. In most cases, a full recovery can be achieved through the improvement of compensation^13,20^. In the present study, N accumulation of maize was inhibited at co-growth period due to N competition from pea plants. However, such a competition may also stimulate the compensation of subdominant species^18^. In this research, expanding maize to pea strip width ratio and lowering N fertilizer rate not only intensified N competition but also improved N compensation of maize plants. Compared to Int-1, the N compensation effect of Int-2 was increased by an average of 9.6% since 75 days after maize sowing. Compared to higher N fertilizer rate at 450 kg N ha^−1^, the N compensation effect with lowered N rate (i.e. 300 kg N ha^−1^) was increased by an average of 6.4% since 75 days after maize sowing. The possible mechanism on improved N compensation due to strip width ratio expansion was assumed as following: (1) the strip width ratio expansion promoted intraspecific competition of maize plants in inner rows to border rows; (2) the border plants also received heavy repression from component pea; (3) the potential for recovery of those border row plants were greatly improved, and (4) those maize plants quickly stretched their leaves and roots to pea strip once pea was harvested.

### Mechanisms on higher N use efficiency and productivity

Nitrogen is required in a larger amount than some other nutrients in crop production^33^. Multiple planting of crops is considered an efficient way to optimize N use and accelerate N concentration in plant tissues^7,34^. In intercropping, component crops with different genotype, physiological metabolism, and morphological traits beneficially improved N use^35,36^, especially in cereal-legume intercropping^37–39^. In the present study, intercropping pea with maize significantly increased the N accumulation in pea plants compared to sole pea. The basic reason might be cereal plants neighboring with legume could facilitate N_2_ fixation, which contributed to final N accumulation^30,40^. In addition, intensifying competition between cereal and legume could promote the legume to fix more atmospheric N^22,41^. As a result, the N accumulation of pea in Int-2 was increased by 5.7% compared to Int-1. Furthermore, intensifying N competition also improved N absorption from applied N fertilizer and enhanced the accumulated N to form more grains. Therefore, the apparent N recovery and N utilization efficiency in Int-2 was increased by 8.0 and 8.9% compared to Int-1, respectively. As a consequence, the grain yield of pea in Int-2 was increased by an average of 11.9% compared to Int-1.

In cereal-legume intercropping, cereal crop accumulated more N in plant tissues than that in sole cropping, indicating there is an N transfer from component strip^42,43^. Commonly, a direct N transfer between intercrops happens during co-growth period^44^; while, an indirect N transfer happens when later-maturating crop independently growing^22,45^. After pea harvest, maize received N compensation due to indirect N transfer from pea strips during its independent growth period. Therefore, the N accumulation of intercropped maize was increased by 31.0% compared to sole maize. In addition, Int-2 increased N accumulation of maize by 5.5% compared to Int-1, as N compensation effect was improved. The improved N compensation effect also promoted N absorption from applied N fertilizer and enhanced the accumulated N to form more grains. Consequently, the apparent N recovery, N utilization efficiency and grain yield of maize in Int-2 was increased by 6.8, 5.1 and 7.7% compared to Int-1, respectively.

In intercropping, legume crop competed severely for soil available N with component cereal at low soil N conditions^17^. In this study, reducing N fertilizer rate enhanced N competition by 6.1%, and thereby increased the total N accumulation as those obtained with a high N rate. Besides, lowered N application also increased apparent N recovery and N utilization efficiency due to intensified N competition. Compared to high N rate, the apparent N recovery and N utilization efficiency of pea with lowered N rate was improved by 10.0 and 6.0%. As a result, the grain yield of pea with lowered N rate was increased as those obtained with a high N rate. For intercropped maize, lowering N rate decreased N accumulation at pea harvest, but had no significant difference with high N rate at maturity. This was mainly attributed to the increased N compensation at maize recovery stage^13,16^. Furthermore, the increase of N compensation also improved apparent N recovery (by 9.7%) and N utilization efficiency (by 11.5%) of maize. Hence, grain yield of maize with lowered N rate was increased as those obtained with a high N rate.

## Conclusions

The present study evaluated the N competition between the two intercrops and assessed the advantages of N compensation and N use efficiency using quantitative analyses. Overall, expanding the maize to pea strip width ratio intensified N competition by 5.2% compared to local strip width ratio. The simple expansion of strip width ratio increased total N accumulation in intercropped pea by 5.7%, apparent N recovery by 8.0%, and improved N utilization efficiency by 8.9% and grain yield by 11.9% compared to the Int-1 system. Expanding strip width ratio also enhanced N compensation of intercropped maize, which promoted total N accumulation by 5.5%, apparent N recovery by 6.8%, N utilization efficiency by 5.1%, and grain yield by 7.7% compared to Int-1. Furthermore, lowering N fertilization promoted apparent N recovery and N utilization efficiency for both pea and maize. These results clearly demonstrated that increasing N complementary use by intercropped pea and maize through modification of strip width ratio and N fertilizer rate can promote N use efficiency and increase productivity of maize/pea intercropping.

## Supplementary information

Supplementary Information.

## References

[1] Branca G, Lipper L, McCarthy N, Jolejole MC (2013). Food security, climate change, and sustainable land management. A review. Agrono. Sustain. Dev..

[2] Chen X, Cui Z, Fan M, Vitousek P, Zhao M, Ma W, Wang Z, Zhang W, Yan X, Yang J, Deng X, Gao Q, Zhang Q, Guo S, Ren J, Li S, Ye Y, Wang Z, Huang J, Tang Q, Sun Y, Peng X, Zhang J, He M, Zhu Y, Xue J, Wang G, Wu L, An N, Wu L, Ma L, Zhang W, Zhang F (2014). Producing more grain with lower environmental costs. Nature.

[3] Tilman D, Fargione J, Wolff B, D'Antonio C, Dobson A, Howarth R, Schindler D, Schlesinger WH, Simberloff D, Swackhamer D (2001). Forecasting agriculturally driven global environmental change. Science.

[4] Huang Y, Tang Y (2010). An estimate of greenhouse gas (N_2_O and CO_2_) mitigation potential under various scenarios of nitrogen use efficiency in Chinese croplands. GCB Bioenergy.

[5] Gan YT (2014). Improving farming practices reduces the carbon footprint of spring wheat production. Nat. Commun..

[6] Cameron KC, Di HJ, Moir J (2013). Nitrogen losses from the soil/plant system: A review. Ann. Appl. Biol..

[7] Tilman D, Cassman KG, Matson PA, Naylor R, Polasky S (2002). Agricultural sustainability and intensive production practices. Nature.

[8] Lithourgidis AS, Dordas CA, Damalas CA, Vlachostergios DN (2011). Annual intercrops: An alternative pathway for sustainable agriculture. Aust. J. Crop Sci..

[9] Tsubo M, Walker S, Mukhala E (2001). Comparisons of radiation use efficiency of mono-/inter-cropping systems with different row orientations. Field Crop Res..

[10] Li L, Sun J, Zhang F, Guo T, Bao X, Smith FA, Smith SE (2006). Root distribution and interactions between intercropped species. Oecologia.

[11] Li L, Sun J, Zhang F, Li X, Yang S, Rengel Z (2001). Wheat/maize or wheat/soybean strip intercropping: I. Yield advantage and interspecific interactions on nutrients. Field Crop Res..

[12] Brooker RW, Karley AJ, Newton AC, Pakeman RJ, Schöb C (2016). Facilitation and sustainable agriculture: A mechanistic approach to reconciling crop production and conservation. Funct. Ecol..

[13] Zhang F, Li L (2003). Using competitive and facilitative interactions in intercropping systems enhances crop productivity and nutrient-use efficiency. Plant Soil.

[14] Li QZ, Sun JH, Wei XJ, Christie P, Zhang FS, Li L (2010). Overyielding and interspecific interactions mediated by nitrogen fertilization in strip intercropping of maize with faba bean, wheat and barley. Plant Soil.

[15] Klimek-Kopyra A, Zaja¸c T, Re¸bilas K (2013). A mathematical model for the evaluation of cooperation and competition effects in intercrops. Eur. J. Agron..

[16] Li L, Yang SC, Li XL, Zhang FS, Christie P (1999). Interspecific complementary and competitive interactions between intercropped maize and faba bean. Plant Soil.

[17] Bedoussac L, Justes E (2011). A comparison of commonly used indices for evaluating species interactions and intercrop efficiency: Application to durum wheat–winter pea intercrops. Field Crop Res..

[18] Hu F, Gan Y, Chai Q, Feng F, Zhao C, Yu A, Mu Y, Zhang Y (2016). Boosting system productivity through the improved coordination of interspecific competition in maize/pea strip intercropping. Field Crop Res..

[19] Andersen M, Hauggaard-Nielsen H, Weiner J, Jensen E (2007). Competitive dynamics in two- and three-component intercrops. J. Appl. Ecol..

[20] Li L, Sun J, Zhang F, Li X, Rengel Z, Yang S (2001). Wheat/maize or wheat/soybean strip intercropping: II. Recovery or compensation of maize and soybean after wheat harvesting. Field Crop Res..

[21] Chai Q, Qin A, Gan Y, Yu A (2013). Higher yield and lower carbon emission by intercropping maize with rape, pea, and wheat in arid irrigation areas. Agrono. Sustain. Dev..

[22] Hu F, Zhao C, Feng F, Chai Q, Mu Y, Zhang Y (2017). Improving N management through intercropping alleviates the inhibitory effect of mineral N on nodulation in pea. Plant Soil.

[23] FAO/UNESCO. Soil Map of the World: Revised Legend/prepared by the Foodand Agriculture Organization of the United Nations. UNESCO (1988).

[24] Gan YT, Siddique KHM, Turner NC, Li XG, Niu JY, Yang C, Liu LP, Chai Q (2013). Ridge-furrow mulching systems-an innovative technique for boosting crop productivity in semiarid rain-fed environments. Adv. Agron..

[25] Yin W, Chen G, Feng F, Guo Y, Hu F, Chen G, Zhao C, Yu A, Chai Q (2017). Straw retention combined with plastic mulching improves compensation of intercropped maize in arid environment. Field Crop Res..

[26] Willey RW, Rao MRA (1980). Competitive ratio for quantifying competition between intercrops. Exp. Agric..

[27] Fageria NK, Baligar VC (2005). Enhancing nitrogen use efficiency in crop plants. Adv. Agron..

[28] Malézieux E, Crozat Y, Dupraz C, Laurans M, Makowski D, Ozier-Lafontaine H, Rapidel B, de Tourdonnet S, Valantin-Morison M (2009). Mixing plant species in cropping systems: Concepts, tools and models. A review. Agrono. Sustain. Dev..

[29] Gómez-Rodríguez O, Zavaleta-Mejía E, González-Hernández VA, Livera-Muñoz M, Cárdenas-Soriano E (2003). Allelopathy and microclimatic modification of intercropping with marigold on tomato early blight disease development. Field Crop Res..

[30] Corre-Hellou G, Fustec J, Crozat Y (2006). Interspecific competition for soil N and its interaction with N_2_ fixation, leaf expansion and crop growth in pea–barley intercrops. Plant Soil.

[31] Hauggaard-Nielsen H, Ambus P, Jensen ES (2003). The comparison of nitrogen use and leaching in sole cropped versus intercropped pea and barley. Nutr. Cycl. Agroecosys..

[32] Andersen M, Hauggaard-Nielsen H, Ambus P, Jensen E (2004). Biomass production, symbiotic nitrogen fixation and inorganic N use in dual and tri-component annual intercrops. Plant Soil.

[33] Hou Z, Li P, Li B, Gong J, Wang Y (2007). Effects of fertigation scheme on N uptake and N use efficiency in cotton. Plant Soil.

[34] Ghosh PK, Mohanty M, Bandyopadhyay KK, Painuli DK, Misra AK (2006). Growth, competition, yields advantage and economics in soybean/pigeonpea intercropping system in semi-arid tropics of India. II. Effect of nutrient management. Field Crop Res.

[35] Li SX, Wang ZH, Hu TT, Gao YJ, Stewart BA (2009). Nitrogen in dryland soils of China and its management. Adv. Agron..

[36] Hardarson G, Zapata F, Danso SKA (1984). Effect of plant genotype and nitrogen fertilizer on symbiotic nitrogen fixation by soybean cultivars. Plant Soil.

[37] Li C (2014). The dynamic process of interspecific interactions of competitive nitrogen capture between intercropped wheat (*Triticum aestivum* L.) and Faba Bean (*Vicia faba* L.). PLoS ONE.

[38] Hauggaard-Nielsen H, Jensen ES (2001). Evaluating pea and barley cultivars for complementarity in intercropping at different levels of soil N availability. Field Crop Res..

[39] Herridge DF, Peoples MB, Boddey RM (2008). Global inputs of biological nitrogen fixation in agricultural systems. Plant Soil.

[40] Boucher DH, Espinosa MJ (1982). Cropping system and growth and nodulation responses of beans to nitrogen in Tabasco, Mexico. Trop. Agric..

[41] Jensen ES (1996). Grain yield, symbiotic N_2_ fixation and interspecific competition for inorganic N in pea-barley intercrops. Plant Soil.

[42] Gooding MJ, Kasyanova E, Ruske R, Hauggaard-Nielsen H, Jensen ES, Dahlmann C, Fragstein P, Dibet A, Corre-Hellou G, Crozat Y (2007). Intercropping with pulses to concentrate nitrogen and sulphur in wheat. J. Agric. Sci..

[43] Rusinamhodzi L, Murwira HK, Nyamangara J (2006). Cotton–cowpea intercropping and its N_2_ fixation capacity improves yield of a subsequent maize crop under Zimbabwean rain-fed conditions. Plant Soil.

[44] Xiao Y, Li L, Zhang F (2004). Effect of root contact on interspecific competition and N transfer between wheat and fababean using direct and indirect ^15^N techniques. Plant Soil.

[45] Jamont M, Piva G, Fustec J (2013). Sharing N resources in the early growth of rapeseed intercropped with faba bean: Does N transfer matter?. Plant Soil.

