# Supplementary material for: Strip width ratio expansion with lowered N fertilizer rate enhances N complementary use between intercropped pea and maize
Source: Sci Rep. 2020 Nov 17;10:19969. doi: 10.1038/s41598-020-76815-7 (PMC7673023; doi:10.1038/s41598-020-76815-7)
Supplement: Supplementary file 1 — Supplementary Information. [file 41598_2020_76815_MOESM1_ESM.doc]

**Strip width ratio expansion with lowered N fertilizer rate enhances N complementary use between intercropped pea and maize**

Falong Hu^1, 2 a^, Yan Tan^3 a^, Aizhong Yu^1, 2^, Cai Zhao^1, 2^, Zhilong Fan^1, 2^, Wen Yin^1, 2^, Qiang Chai^1, 2*^, Weidong Cao^4*^

^1^ Gansu Provincial Key Laboratory of Arid Land Crop Science, Lanzhou 730070, China

^2^ College of Agronomy, Gansu Agricultural University, Lanzhou 730070, China

^3^ College of Forestry, Gansu Agricultural University, Lanzhou 730070, China

^4^ Institute of Agricultural Resources and Regional Planning, Chinese Academy of Agricultural Sciences, Beijing 100081, China

^a^ Contributed equally to the work

^*^ Corresponding author. Email: chaiq@gsau.edu.cn; caoweidong@caas.cn

Phone: +86 931 763-1167

1. **The three years data of nitrogen competitive ratio (N-CR)**


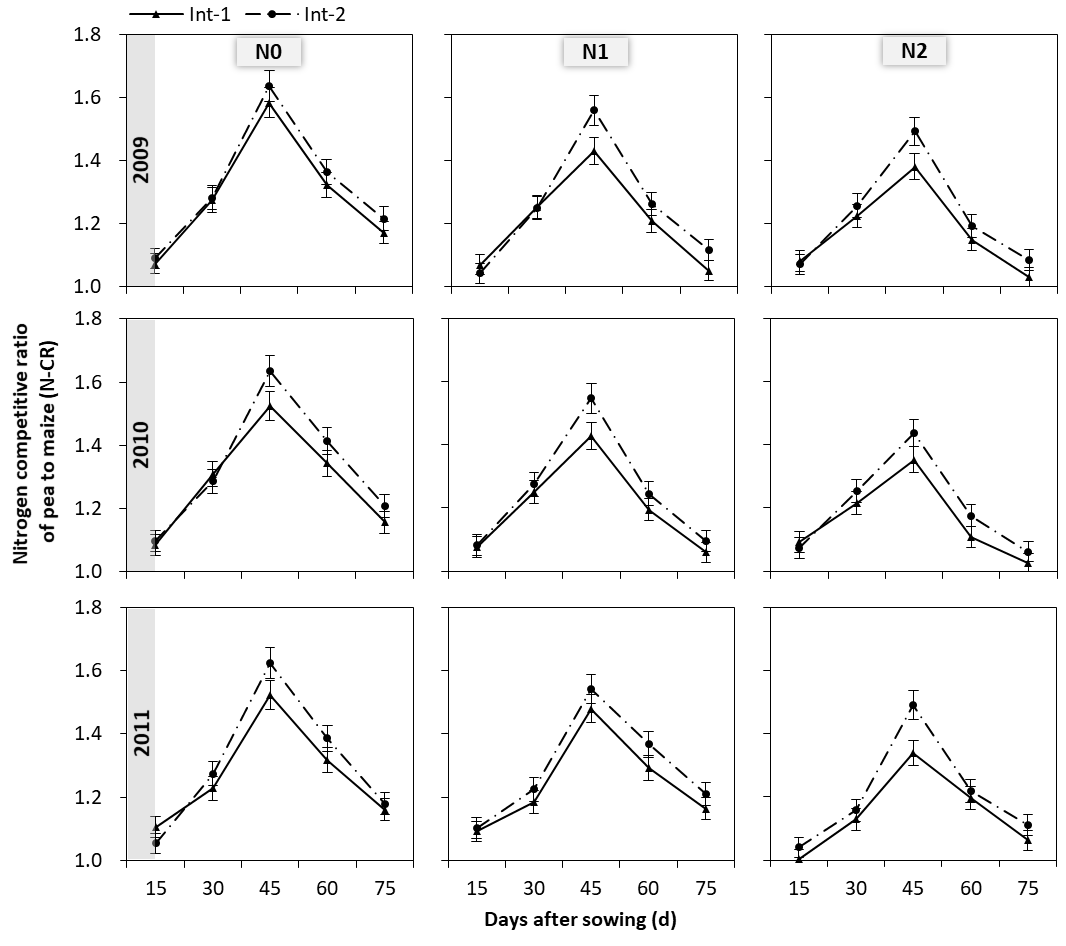


**2. The three years data of apparent nitrogen recovery (ANR)**

| Cropping pattern ^a^ | N rate ^b^ | 2009 | |  | 2010 | |  | 2011 | |
| --- | --- | --- | --- | --- | --- | --- | --- | --- | --- |
|  |  | Pea | Maize |  | Pea | Maize |  | Pea | Maize |
| Sole cropping | N1 | 33.7 | 27.2 |  | 32.2 | 28.5 |  | 33.3 | 29.4 |
|  | N2 | 30.6 | 25.3 |  | 30.1 | 26.7 |  | 31.1 | 27.4 |
| Intercropping | N1 | 39.2 | 31.8 |  | 36.1 | 31.7 |  | 39.8 | 32.3 |
| (Int-1) | N2 | 35.4 | 29.2 |  | 32.4 | 29.4 |  | 36.9 | 30.1 |
| Intercropping | N1 | 44.7 | 34.3 |  | 46.3 | 35.3 |  | 45.9 | 35.5 |
| (Int-2) | N2 | 36.1 | 30.9 |  | 39.2 | 31.6 |  | 38.7 | 31.9 |
| Significance (*p* value) | |  |  |  |  |  |  |  |  |
| Year (Y) | | NS | 0.024 |  | - | - |  | - | - |
| Cropping system (C) | | NS | <0.001 |  | 0.010 | <0.001 |  | 0.024 | <0.001 |
| N rate (N) | | NS | <0.001 |  | NS | 0.002 |  | NS | <0.001 |
| C × N | | <0.001 | NS |  | NS | <0.001 |  | <0.001 | <0.001 |
| Y × C × N | | NS | NS |  | - | - |  | - | - |

**3. The three years data of nitrogen utilization efficiency (NutE)**

| Cropping pattern ^a^ | N rate ^b^ | 2009 | |  | 2010 | |  | 2011 | |
| --- | --- | --- | --- | --- | --- | --- | --- | --- | --- |
|  |  | Pea | Maize |  | Pea | Maize |  | Pea | Maize |
| Sole cropping | N0 | 11.8 | 42.6 |  | 13.5 | 44.0 |  | 14.3 | 41.1 |
|  | N1 | 15.5 | 43.0 |  | 15.5 | 45.5 |  | 15.9 | 42.6 |
|  | N2 | 14.3 | 42.9 |  | 15.6 | 44.6 |  | 15.1 | 37.6 |
| Intercropping | N0 | 12.2 | 45.8 |  | 14.6 | 45.8 |  | 14.1 | 45.8 |
| (Int-1) | N1 | 16.6 | 46.2 |  | 17.5 | 47.2 |  | 15.1 | 47.0 |
|  | N2 | 15.2 | 42.2 |  | 16.0 | 42.7 |  | 14.9 | 41.7 |
| Intercropping | N0 | 13.3 | 47.9 |  | 14.2 | 49.8 |  | 17.0 | 49.1 |
| (Int-2) | N1 | 17.3 | 48.6 |  | 17.9 | 49.3 |  | 18.4 | 49.1 |
|  | N2 | 16.3 | 44.1 |  | 16.3 | 43.4 |  | 17.4 | 43.8 |
| Significance (*p* value) | |  |  |  |  |  |  |  |  |
| Year (Y) | | <0.001 | <0.001 |  | - | - |  | - | - |
| Cropping system (C) | | 0.002 | <0.001 |  | 0.008 | <0.001 |  | <0.001 | <0.001 |
| N rate (N) | | <0.001 | <0.001 |  | <0.001 | <0.001 |  | NS | <0.001 |
| C × N | | NS | <0.001 |  | NS | <0.001 |  | NS | NS |
| Y × C × N | | NS | NS |  | - | - |  | - | - |
